# Long-Term Results after Bilateral Sacrospinous Colposuspension: A Prospective Study

**DOI:** 10.3390/jcm12144691

**Published:** 2023-07-14

**Authors:** Gautier Chene, Emanuele Cerruto, Stephanie Moret, Erdogan Nohuz

**Affiliations:** 1 Department of Gynecology, Hôpital Femme Mère Enfant (HFME), 59 Boulevard Pinel, University Hospital of Lyon, 69500 Bron, France; cerrutoemanuele@gmail.com (E.C.); stephanie.moret@chu-lyon.fr (S.M.); erdogan.nohuz@chu-lyon.fr (E.N.); 2 EMR 3738 CICLY, University Claude Bernard of Lyon 1, 69000 Lyon, France

**Keywords:** sacrospinous ligament, Richter, sacrospinous colpopexy, pelvic organ prolapse, vaginal vault prolapse, vaginal mesh

## Abstract

The loss of apical support is usually present in patients with pelvic organ prolapse. An effective correction for the vaginal apex may be an essential part of a durable repair for these women. Apical suspension of the sacrospinous ligament is likely one of the best treatments by the vaginal route. We proposed the evaluation of the functional and anatomical long-term results of an ultralight and macroporous sling. In this prospective study, bilateral sacrospinous colposuspension was performed in 32 patients with a specific mesh. Functional assessment with several validated quality of life questionnaires and pelvic examination was performed at 1, 6, 12, and 24 months after surgery. Pelvic examination using the POP-Q classification showed a very good efficacy of the BSC mesh with only three prolapse recurrences at 24 months after surgery. All the following QoL scores were significantly improved by two years: PFIQ-7 (*p* < 0.0001), PFDI-20 (*p* < 0.0001), and SF-12 (*p* < 0.0001). No improvement was achieved by the PISQ12 questionnaire. This vaginal minimally invasive procedure is effective, quick, reproducible, and easy. It may be a relevant option for a vaginal vault or cervical or uterine prolapse.

## 1. Introduction

Apical vaginal prolapse often occurs as a site of pelvic organ prolapse, especially in severe and advanced prolapse cases. It is also demonstrated that apical support is essential during surgical correction to limit the risk of medium- and long-term recurrence. Different apical suspension procedures have been described via the vaginal or abdominal route. The vaginal route is most often chosen in scientific studies (up to 90% of procedures performed by this route) [1,2]. Several vaginal procedures were described that involved suspending the pelvic ligament (including the sacrospinous ligament) with native tissue or surgical mesh. The vaginal Richter’s sacrospinofixation technique is the gold standard for apical prolapse. However, several intraoperative complications have been reported (a risk of vascular injury and a risk of nerve compression up to 17%). The risk of subsequent recurrence of the anterior compartment, especially when the sacrospinofixation is unilateral, may be related to the posterior deflection of the vaginal apex and is estimated to be between 2.4 and 19% [1,2]. Chronic pelvic pain and/or neuropathic pain have also been reported in the literature in 5% of cases and rate of de novo dyspareunia may occur in up to 16% of cases [3,4]. Another surgical option may be the use of a posterior bilateral isthmic sling. Prof. Kieback designed and developed an ultra-light and macroporous polypropylene sling named the BSC-mesh™ (Bilateral Sacrospinous Colposuspension-mesh™, AMI, Feldkich, Austria) which allows a bilateral and medial sacrospinous fixation with the I-Stitch™ device [5]. This vaginal minimally invasive procedure with only a single vaginal incision may be a reliable option as it prevents posterior deviation of the vagina. We propose the evaluation of the functional and anatomical long-term outcomes of the bilateral sacrospinous colposuspension with the BSC-mesh™ in a prospective series. 

## 2. Materials and Methods

A single-center prospective study of women undergoing surgical repair by the BSC-mesh was conducted from September 2019 to March 2023 in accordance with the Declaration of Helsinki and was approved by the Institutional Review Board of “Comité de Protection des Personnes Ile de France III” (protocol code Am8995-2-3695-NI and date of approval: 30 July 2019) for studies involving humans. This study is registered under ClinicalTrials.gov identifier: NCT03911778.

All patients presenting symptomatic stage II or higher apical prolapse according to the Pelvic Organ Prolapse Quantification system (POP-Q) were invited to participate in the study. Consenting women agreed to provide baseline and follow-up data at 1, 6, 12, and 24 months post-operatively on their symptoms, anatomical correction, and quality of life using specific questionnaires. A concomitant anterior and/or posterior colporrhaphy by native tissue could be performed when indicated. Vaginal hysterectomy could be performed only in the case of symptomatic abnormalities visualized on preoperative ultrasound (fibroids and adenomyosis, for example). No other mesh or sling (like TVT or TOT) could be used during the procedure. A pre-operative urodynamic assessment was systematically performed. 

### 2.1. Patient Reported Outcome Measures (PROMs)

The primary objective was to assess the degree of improvement after prolapse surgery using short-, medium-, and long-term health-related quality of life (HRQOL) questionnaires at 1, 6, 12, and 24 months. We used several validated quality-of-life questionnaires:

The Patient Global Impression of Improvement (PGI-I) questionnaire for urogenital prolapse [6]. This is a self-administered and validated questionnaire that provides a global index of response to prolapse surgery. It is a scale from 1 (very great improvement) to 7 (very great deterioration) that describes the current postoperative status compared to the preoperative status. 

The Short Form 12 (SF-12) questionnaire [7]: this is a self-administered, validated global quality of life questionnaire (score from 0 to 100) concerning the physical and mental components of quality of life. The higher the score, the better the quality of life.

The Pelvic Floor Distress Inventory-20 (PFDI-20) questionnaire [8] is a self-administered and validated questionnaire for women with pelvic floor disorders. It measures the extent to which bowel, bladder, and pelvic symptoms bother the patient (from 0 to 100). The higher the score, the worse the quality of life.

The Pelvic Floor Impact Questionnaire-7 (PFIQ-7) questionnaire [8] is a self-administered and validated questionnaire for women with pelvic floor disorders. It measures the extent to which activities, relationships, and feelings have been affected by bladder, bowel, or vaginal symptoms (range 0–300). The higher the score, the worse the quality of life.

The short form of the Pelvic Organ Prolapse/Urinary Incontinence Sexual Questionnaire (PISQ-12) [9] is a self-administered and validated instrument to evaluate sexual function in women with pelvic organ prolapse. It measures three domains: behavioral–emotional, physical, and partner-related. Responses are scored on a five-point Likert scale ranging from 1 to 4. A total of 48 is the maximum score: higher scores indicate better sexual function. 

Pain was evaluated using a 10 cm VAS. 

Secondary objectives included assessing the results of the surgical procedure by clinical examination using the POP-Q classification. Apical prolapse of stage II or higher was considered a failure of the surgical procedure.

### 2.2. Surgical Technique

A preoperative ultrasound was always performed to check the presence or absence of uterine anomalies. All procedures were performed under general anesthesia or locoregional anesthesia by an experienced urogynecological surgeon (GC). The published standardized technical protocol was followed [5,10]. Briefly, the patients were in the lithotomy position. A short longitudinal incision was made on the midline of the posterior vaginal wall followed by bilateral blunt lateral dissection with Metzenbaum scissors and a finger toward the sacrospinous ligament (SSL). One suture was placed on each SSL using the I-Stitch™ instrument. Two sutures were threaded through the BSC mesh™ for medial fixation (either on the isthmic wall or on the vaginal apex in case of hysterectomy). The BSC mesh™ (AMI, Feldkich, Austria) is an ultralight monofilament polypropylene U-shaped isoelastic mesh. I-Stitch™ sutures were tied through the arms of the mesh and the knot was guided with the finger to the SSL. An additional anterior or posterior colporrhaphy [11] could be performed and the vaginal incision was finally closed. The urinary catheter and vaginal gauze were placed and removed on the second postoperative day.

The surgeon rated the complexity of the procedure using a 10-cm VAS scale ranging from “easy” (10) to “very difficult” (0) immediately after surgery. The duration of the operation and the perioperative and postoperative complications, using the Clavien–Dindo classification [12], a validated scoring system for surgical complications, were also reported. 

### 2.3. Statistical Analysis

Statistical analysis was performed using the McNemar test and Student’s *t*-test for the paired series. Data are described by their mean and standard deviation for continuous quantitative data and by their number and frequency for qualitative data.

Statistical analysis was performed using SAS software (SAS Studio 3.6; SAS Institute Inc., Cary, CO, USA). A *p*-value of less than 0.05 was considered significant.

### 2.4. Sample Size Calculation

Based on previous studies of sacrospinofixation with a strip [13,14,15] which found a rate of satisfied patients of 96 to 98% [13,14,15] and a rate of patients with symptom improvement based on the PGI-I score of 96% [5], the hypothesis in this study was to observe patients with a rate of symptom improvement of 98%. With a margin of error of 5%, an alpha risk of 5%, and an error rate of 5%, the number of subjects required was 31 patients. 

Taking into account a risk of approximately 5% of patients leaving the study prematurely, a total of 33 patients were included.

## 3. Results

### 3.1. Surgical Outcomes

**Figure 1:**
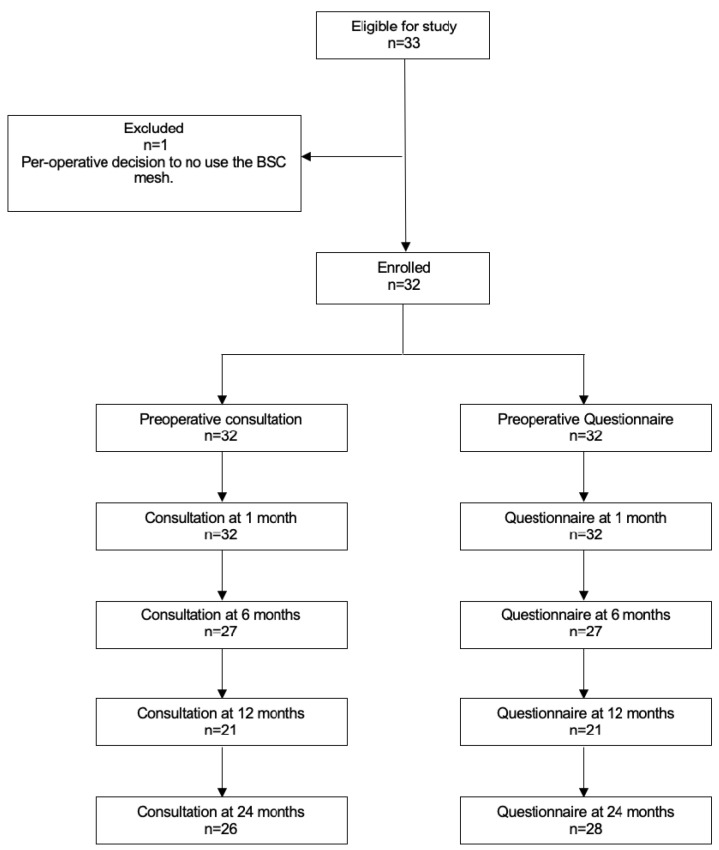
Study Flowchart.

A total of 33 patients were eligible, 32 were enrolled, and 1 was excluded (decision not to use the BSC mesh™ during the surgical procedure) (Figure 1). Patient characteristics and intraoperative data are presented in Table 1. The mean age was 65 years (±2.4). The majority of patients were postmenopausal. BSC mesh™ placement was rapid (mean time: 11.7 min ± 1.8) and easy (9.6 ± 0.1 on EVA) (Table 2). Concomitant anterior colporrhaphy was performed in 18 patients (56.2%) and concurrent posterior colporrhaphy was performed in 11 patients (34.4%) [11]. The estimated blood loss was 68.1 ± 10.5 mL. There was no per-operative complication.

There were two Clavien–Dindo grade 2 postoperative complications: namely two cases of urinary tract infection. The VAS pain scale on the first day was 2.4 ± 0.2. Patients mostly went home between the second and the third postoperative day. 

At 1 month, 32 patients (100%) had a clinical examination with anatomical assessment and answered the different questionnaires. At 6 months, 27 patients had a clinical examination with anatomical assessment and answered the questionnaires. At 12 months, 21 patients had a clinical examination with anatomical assessment and answered the questionnaires. Lastly, at 24 months, 26 patients had a clinical examination with anatomical assessment and 28 patients answered the questionnaires. 

Pelvic examination using the POP-Q classification showed a very good efficacy of the BSC mesh™. Anatomical evaluation for the apical compartment was the following for C point (Table 3): C point at −6.1 ± 0.5 (*p* < 0.0001) one month after surgery; C point at −5.7 ± 0.6 (*p* < 0.0001) three months after surgery; C point at −5.5 ± 0.4 (*p* < 0.0001) 12 months after surgery; and C point at −6.2 ± 0.1(*p* < 0.0001) 24 months after surgery. Three patients had prolapse recurrences (two apical prolapses and one cystocele): one patient required a laparoscopic sacrocolpopexy, one patient needed a conventional unilateral sacrospinofixation, and one patient had an anterior colporrhaphy [11]. In total, six patients had a stress urinary incontinence treatment (five bulking agent injections, one TOT) [16].

There was a statistically non-significant decrease in the rate of SUI or urinary urgency. Dysuria was significantly improved thanks to prolapse correction. There was no erosion nor mesh shrinkage at 24 months (Table 4).

### 3.2. Patient Outcomes

According to the global index PGI-I, there was a statistically significant improvement at 1, 6, 12, and 24 months: 31 (31/32 = 96.9%) patients felt improved at 1 month, 24 (24/27 = 88.9%) patients at 6 months, 18 (18/21 = 85.7%) at 12 months, and 24 (24/28 = 85.7%) at 24 months. 

All the following QoL scores were significantly improved by 2 years: PFIQ-7 (*p* < 0.0001), PFDI-20 (*p* < 0.0001), and SF-12 (*p* < 0.0001). There was no improvement with the PISQ12 questionnaire (Table 5).

## 4. Discussion

**Figure 2:**
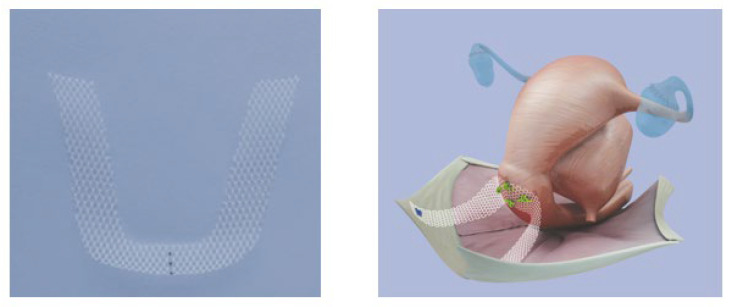
The BSC Mesh™ is designed to establish symmetrical and bilateral suspension of the vaginal vault from the sacrospinous ligament. Courtesy of AMI, Austria.

This prospective series demonstrates very good anatomical and functional efficacy of sacropinofixation using the BSC mesh™ (Figure 2). As stated by Prof. Kieback who designed the BSC mesh™ [5], bilateral correction allows symmetrical suspension of the vaginal apex and the creation of neo-ligaments inserting on the sacrospinous ligament. In comparison with the unilateral Richter’s sacrospinofixation where there may be a recurrence of the anterior compartment mainly related to the deviation of the vaginal axis, the BSC mesh™ keeps the vagina in a more horizontal plane closer to the original anatomic position [17]. We did not record any hematoma or hemorrhagic complications since there is no extensive dissection of the pararectal fossa with this vaginal minimally invasive technique. Moreover, several minimally invasive suture devices and anchoring systems were developed to reduce dissection of the pararectal fossa and the sacrospinous ligament (only finger-guided dissection and placement of sutures): the I-Stitch™ (AMI, Feldkirch, Austria), the Capio slim™ (Boston Scientific, Malborough, MA, USA), the applicator Anchorsure™ (Neomedic International, Terrassa, Spain), and the EnPlace device™ (Lina, Root D4, Lucerne, Switzerland). The BSC mesh™ is therefore easily placed with the I-Stitch™ instrument with minimal dissection. We did not observe any vaginal erosion or mesh shrinkage. Vaginal mesh erosion is one of the main complications following the use of surgical mesh devices to repair pelvic organ prolapse. The absence of erosion is likely related to the characteristics of the mesh (ultra-lightweight: 21 g/m^2^, monofilament, macroporous with pore size >1.9 mm, and 93% porosity) leading to rapid anatomical integration and a reduced foreign body tissue reaction. Indeed, a preliminary histopathological analysis of a BSC mesh™ (a case of total hysterectomy in a patient with the BSC mesh™) reported minimal body foreign reaction with “limited fibrosis around each individual mesh fiber without confluence” [18]. Larger prospective multicenter cohorts are still needed to confirm outcomes of our study. It is also notable that exposure to non-absorbable threads has been reported with classical Richter’s sacrospinofixation [4]. Another technique for the treatment of prolapse of the vaginal apex using a mesh was previously developed: the posterior Intravaginal Slingplasty (the IVS technique) procedure involved a synthetic mesh placed through the ischiorectal space and iliococcygeus muscle on both sides to the level of the vaginal vault [19,20]. However, several significant severe complications following IVS procedures were reported including rectal injuries (0.15% in the systematic review by Feiner et al. and 1% in the Austrian registry), tape exposures (8% in the systematic review by Feiner et al. and 8.7% in the Austrian registry), and tape-related abscess (respectively, 0.15% in the systematic review by Feiner et al. and 0.2% in the Austrian registry) [19,20]. This technique is no longer available. We likely do not have any of these adverse effects with the BSC mesh™ procedure due to the type and biomechanical properties of the mesh and the surgical technique. All quality of life scores except for the sexuality questionnaire were improved. There may be several explanations: sexual satisfaction is multi-factorial and is not limited to an anatomical correction of a prolapse. Indeed, the population studied was older and probably less sexually active than a younger population would be. Nevertheless, the PISQ 12 scores remain stable over time suggesting that BSC Mesh™ does not alter sexuality. 

The anatomical success rate (from 80 to 100%) with the classical Richter’s sacrospinofixation is well known but severe neurovascular complications related to extensive dissection difficulties have been reported [1,2,3,4]. Using a minimally invasive surgical procedure may be safer since there is a limited dissection of the pararectal fossa. Moreover, the good anatomical outcome with the BSC mesh™ may be related to the bilateral fixation whereas classical Richter’s sacrospinofixation is often unilateral. Our results are consistent with those of the literature:

Hemtenmacher et al. [18] reported a series of 132 patients with a 6-month follow-up: there was no recurrence of prolapse at 6 months for the 56 patients (56/132 = 42%) following the invitation for the physical examination at 6 months. Neither rectal injury nor mesh erosion was observed. The rate of dyspareunia was unchanged with 6% of patients in comparison with preoperative data. The authors highlighted the potential effect of the BSC mesh™ to improve SUI and/or urge disorders. We have similar results in our study. Other prospective studies specifically designed for urinary disorders are still needed. Lastly, the authors explained the good efficacy of the BSC mesh™ procedure by the biocompability and the biomechanical properties of the mesh. They stated that the BSC mesh™ closely mimics the original anatomy of the uterosacral ligaments recreating the physiological pelvic anatomy. 

In a retrospective study of 30 patients with a 1-year follow-up, Hosni et al. [17] found that the POP-Q parameters were significantly improved at twelve months after surgery compared to the baseline. The rate of anatomical success at one year was 100%. No intraoperative adverse effects were recorded. There were two postoperative urinary retentions with spontaneous remission thanks to two days of additional urinary catheterization and the use of an alpha-blocker. No visceral injury nor mesh erosion was observed. None of the patients complained of dyspareunia. Urinary symptoms were also improved but this is owing firstly to the fact that the cohort is small and second because an anterior colporrhaphy was performed in most patients (87%); it seems difficult to make a correlation with the BSC mesh™ procedure. 

Another study confirmed a significant improvement in the quality of life scores at 12 months [21]. Unfortunately, the study was not designed to assess anatomical outcomes.

Last but not least, the results in 53 patients at 30 months of bilateral sacrospinofixation using either another manufacturer’s mesh or a posterior mesh cut by the surgeon himself were reported by Capmas et al. [22]: four women (7.5%) had a prolapse recurrence. Women’s satisfaction level was high (8/10 on a visual analog scale). No rectal nor vesical injuries were reported. Mesh erosion was observed in 8.7% of patients. Nevertheless, only 1.9% needed a mesh removal whereas other patients were successfully treated with local vaginal estrogen therapy. Mesh shrinkage was present in 6.5% of cases. Dyspareunia was noted in 3.7% of patients. Postoperative urinary disorders were not evaluated because of the concomitant associated surgery using a suburethral sling in 32.1% of cases. We and previous studies pertaining to the BSC mesh™ did not share the same adverse events. We really think that a specifically designed mesh for apical prolapse repair should be used and not a homemade mesh (outcomes related to the specific mesh in comparison with the posterior mesh cut by the surgeon himself were not reported in the study by Capmas et al.) [22]. 

Surgical treatment for women with an apical vaginal prolapse was evaluated by the Cochrane Database Group in 2016 [1]: recurrence may occur between 1 to 7% of cases after surgery with mesh by the vaginal route. However, most of the evaluated transvaginal meshes are no longer available and there is a lack of studies with new lighter meshes. The authors stated that no clear conclusion could be reached.

A limitation of the present study is the lack of a comparative group (such as conventional Richter’s sacrospinofixation with native tissue). The strengths include the fact that this is the first prospective study with long-term functional and anatomical results. We also selected several validated QoL questionnaires to assess all components of the quality of life. 

Since the FDA ordered post market surveillance studies by manufacturers of urogynecologic mesh to address specific safety and effectiveness concerns, many devices for the transvaginal repair of pelvic organ prolapse are no longer commercially available; conventional transvaginal native tissue repair comes back [23,24]. However, there may be more recurrence rates with native tissue repair [17]. Vaginal minimally invasive standardized techniques with new ultralight mesh should be considered as a relevant option. 

## Figures and Tables

**Table 1:** Baseline characteristics of patients (n = 32).

Age	65.0 ± 2.4
BMI kg/m^2^, mean ± SD	27.2 ± 0.9
Parity	3.0 ± 0.3
Number of vaginal deliveries	2.6 ± 0.3
Menopausal status, n (%)	27 (84.4)
Previous hysterectomy n (%)	9 (28.1)
Urinary stress incontinence n (%)	11 (34.4)
grade I	4
grade II	5
grade III	2
Urinary urgency n (%)	10 (31.2)
Dysuria n (%)	18 (56.2)
Preoperative UDA ^a^ n (%)	32 (100)
mean closure pressure (cm H_2_O)	61.7 ± 5.9
maximum flow (mL/s)	25.0 ± 2.7

Data are mean (±standard deviation) or n (%); ^a^ UDA: urodynamic assessment.

**Table 2:** Perioperative outcomes (n = 32).

Operative time min (SD)	76.4 ± 4.4
Mean (SD) BSC Mesh placement duration min (SD)	11.7 ± 1.8
Concomitant vaginal hysterectomy n (%)	2 (6.2)
Concomitant anterior colporrhaphy n (%)	18 (56.2)
Concomitant posterior colporrhaphy n (%)	11 (34.4)
Estimated blood loss mL (SD)	68.1 ± 10.5
Complexity of the procedure ^a^ (VAS scale)	9.6 ± 0.1
Fever (≥38 °C)	0 (0.0)
Hemorrhage	0 (0.0)
Urinary infection	2 (6.2)
Pelvic infection	0 (0.0)
D−1 VAS pain scale	2.4 ± 0.2
Hospital stay (days)	2.9 ± 0.2

Data are mean (±standard deviation) or n (%); ^a^ The surgeon rated the complexity of the procedure using a 10-cm VAS scale ranging from “easy” (10) to “very difficult” (0) immediately after surgery.

**Table 3:** Anatomical results according to their POP-Q classification.

	Preoperative n = 32	1 Month n = 32	p*	6 Months n = 27	p*
Aa (cm)	1.4 ± 0.3	−2.5 ± 0.1	<0.0001	−2.2 ± 0.1	<0.0001
Ba (cm)	1.4 ± 0.3	−2.7 ± 0.1	<0.0001	−2.4 ± 0.1	<0.0001
C (cm)	2.1 ± 0.1	−6.1 ± 0.5	<0.0001	−5.7 ± 0.6	<0.0001
Ap (cm)	−0.1 ± 0.3	−2.5 ± 0.1	<0.0001	−2.5 ± 0.1	<0.0001
Bp (cm)	−0.2 ± 0.3	−2.7 ± 0.1	<0.0001	−2.6 ± 0.1	<0.0001
D (cm)	−0.03 ± 0.4	−7.4 ± 0.2	<0.0001	−6.4 ± 0.8	<0.0001
tvl (cm)	7.7 ± 0.1	8.0 ± 0.1	0.01	7.7 ± 0.1	0.26
Aa (cm)	1.4 ± 0.3	−2.0 ± 0.2	<0.0001	−2.2 ± 0.1	<0.0001
Ba (cm)	1.4 ± 0.3	−2.2 ± 0.2	<0.0001	−2.4 ± 0.1	<0.0001
C (cm)	2.1 ± 0.1	−5.5 ± 0.4	<0.0001	−6.2 ± 0.1	<0.0001
Ap (cm)	−0.1 ± 0.3	−2.2 ± 0.1	<0.0001	−2.2 ± 0.1	0.0003
Bp (cm)	−0.2 ± 0.3	−2.4 ± 0.1	<0.0001	−2.4 ± 0.1	0.0006
D (cm)	−0.03 ± 0.4	−7.1 ± 0.2	<0.0001	−6.7 ± 0.3	<0.0001
tvl (cm)	7.7 ± 0.1	7.5 ± 0.1	0.59	7.3 ± 0.1	0.09

Data are mean (±standard deviation) or n (%); p*: compared to preoperative data; Tvl: total vaginal length.

**Table 4:** Follow-up.

	Preoperative n = 32	1 Month n = 32	p*	6 Months n = 27	p*
SUI ^a^	11 (34.4)	3 (9.4)	0.02	4 (14.8)	0.01
grade I	4	2		1	
grade II	5	1		2	
grade III	2	0		1	
Urinary urgency	10 (31.2)	2 (6.2)	0.01	2 (7.4)	0.03
Dysuria	18 (56.2)	1 (3.1)	<0.0001	2 (7.4)	0.001
Vaginal mesh exposure		0 (0.0)		0 (0.0)	
Mesh shrinkage		0 (0.0)		0 (0.0)	
Urinary infection		0 (0.0)		0 (0.0)	
Vaginal infection		0 (0.0)		0 (0.0)	
SUI ^a^	11 (34.4)	4 (19.0)	0.12	6 (23.1)	0.51
grade I	4	1		2	
grade II	5	2		4	
grade III	2	1		0	
Urinary urgency	10 (31.2)	5 (23.8)	1.00	4 (15.4)	0.29
Dysuria	18 (56.2)	1 (4.8)	0.01	0 (0.0)	0.001
Vaginal mesh exposure		0 (0.0)		0 (0.0)	
Mesh shrinkage		0 (0.0)		0 (0.0)	
Urinary infection		0 (0.0)		0 (0.0)	
Vaginal infection		0 (0.0)		0 (0.0)	

Data are mean (±standard deviation) or n (%); p*: compared to preoperative data; ^a^ Stress Urinary Incontinence.

**Table 5:** Quality of life scores.

	Preoperative n = 32	1 Month n = 32	p*	6 Months n = 27	p*
PGI-I ^a^		1.8 ± 0.2		2.0 ± 0.2	
PGI-I ^a^ (scores 1,2,3)		31 (96.9)		24 (88.9)	
SF-12					
Mental score	38.4 ± 1.6	48.2 ± 1.5	<0.0001	52.1 ± 1.2	<0.0001
Physical score	41.7 ± 2.0	51.3 ± 1.3	<0.0001	51.7 ± 1.6	0.0003
PFIQ-7 ^b^	116.2 ± 13.9	21.9 ± 7.8	<0.0001	5.1 ± 2.1	<0.0001
UIQ-7	42.1 ± 6.0	7.0 ± 3.2	<0.0001	2.8 ± 1.4	<0.0001
CRAIQ-7	31.1 ± 5.0	8.6 ± 3.3	<0.0001	1.6 ± 1.1	<0.0001
POPIQ-7	43.0 ± 6.1	6.4 ± 2.5	<0.0001	0.7 ± 0.5	<0.0001
PFDI-20 ^c^	143.5 ± 10.1	28.1 ± 7.2	<0.0001	15.4 ± 3.6	<0.0001
POPDI-6	63.8 ± 3.7	4.0 ± 1.6	<0.0001	4.4 ± 1.5	<0.0001
CRADI-8	35.0 ± 4.0	11.7 ± 2.9	<0.0001	4.5 ± 1.4	<0.0001
UDI-6	44.7 ± 5.1	12.4 ± 3.6	<0.0001	6.5 ± 2.3	<0.0001
Global pain ^d^		0.6 ± 0.1		0.3 ± 0.1	
PISQ12 ^e^	27.2 ± 2.3	45.0 ± 1.0	0.44	10.1 ± 1.7	0.03
PGI-I ^a^		2.2 ± 0.3		1.7 ± 0.2	
PGI- I ^a^ (scores 1,2,3)		18 (85.7)		24 (85.7)	
SF-12					
Mental score	38.4 ± 1.6	50.7 ± 1.7	<0.0001	53.3 ± 0.8	<0.0001
Physical score	41.7 ± 2.0	52.4 ± 2.4	0.0005	55.4 ± 1.2	<0.0001
PFIQ-7 ^b^	116.2 ± 13.9	9.1 ± 3.8	<0.0001	7.0 ± 3.4	<0.0001
UIQ-7	42.1 ± 6.0	5.9 ± 2.7	<0.0001	1.0 ± 0.5	<0.0001
CRAIQ-7	31.1 ± 5.0	2.0 ± 1.4	0.0007	3.6 ± 1.9	<0.0001
POPIQ-7	43.0 ± 6.1	1.1 ± 0.9	0.0002	2.4 ± 1.6	<0.0001
PFDI-20 ^c^	143.5 ± 10.1	21.8 ± 6.0	<0.0001	15.0 ± 3.5	<0.0001
POPDI-6	63.8 ± 3.7	5.5 ± 2.1	<0.0001	4.8 ± 1.7	<0.0001
CRADI-8	35.0 ± 4.0	5.6 ± 1.7	<0.0001	3.7 ± 1.3	<0.0001
UDI-6	44.7 ± 5.1	10.6 ± 3.3	<0.0001	6.8 ± 2.0	<0.0001
Global pain ^d^		0.02 ± 0.02		0.1 ± 0.1	
PISQ12 ^e^	27.2 ± 2.3	38.1 ± 2.2	0.07	41.0 ± 1.7	0.17

Data are mean (±standard deviation) or n (%); ^a^ PGI-I: Patient Global Impressions scale—Improvement; ^b^ PFIQ: Pelvic Floor Impact Questionnaire; ^c^ PFDI: Pelvic Floor Distress Inventory; ^d^ Global pain (10 cm VAS); ^e^ PISQ: Pelvic Organ Prolapse/Urinary Incontinence Sexual Questionnaire; p*: compared to preoperative data.

## Data Availability

The datasets used are available from the corresponding author upon reasonable request.
